# Disseminated Gonococcal Infection Due to a Mediport Catheter Infection

**DOI:** 10.7759/cureus.48180

**Published:** 2023-11-02

**Authors:** Ambreen Malik, Michael Peniston, Jason Donaghue, Leonard B Johnson

**Affiliations:** 1 Infectious Diseases, Ascension St. John Hospital, Detroit, USA; 2 Internal Medicine, Ascension St. John Hospital, Detroit, USA

**Keywords:** mediport infection, gonococcal bacteremia, disseminated gonococcal infection, gram-negative bacteremia, disseminated gonorrhea

## Abstract

Gonorrhea is a sexually transmitted disease caused by *Neisseria gonorrhoeae* and has a wide clinical spectrum that can range from asymptomatic to disseminated disease. Most women with gonorrhea are asymptomatic and if left untreated, it can lead to serious complications like pelvic inflammatory disease (PID) and infertility. Disseminated gonococcal infection (DGI) is usually characterized by dermatitis, tenosynovitis, and septic arthritis but rarely can also cause bacteremia, endovascular infections, osteomyelitis, and meningitis. Gonococcal bacteremia is regarded as a disseminated disease and is typically associated with infection of the mucosal surfaces such as the urethra, endocervix, and pharynx. This report, to the best of our knowledge, presents a case of DGI associated with a mediport catheter in a patient with breast cancer without any history of gonococcal symptoms. She was monogamous and denied any history of sexually transmitted infections. The patient presented with fever and chills associated with pain and purulent discharge from the mediport catheter site. The mediport catheter was removed, and antibiotics were initiated. Both blood and wound cultures grew *N. gonorrhoeae*. She completed a 10-day course of ceftriaxone and improved clinically with complete remission of her symptoms. A review of the literature on the reported cases of DGI associated with bacteremia and endovascular infections is also presented.

## Introduction

Gonorrhea is the second most common bacterial sexually transmitted disease in the United States and as per the CDC estimate, infected 1.6 million new cases in 2018. According to CDC surveillance data, the overall rate of reported gonorrhea cases increased by 4.6% during 2020-2021 [1]. *Neisseria gonorrhoeae* can be recovered from a mucosal site in at least 80% of symptomatic patients, highlighting the importance of testing all the mucosal surfaces including the urethra or endocervix, the rectum, and the pharynx, regardless of symptoms or exposure history. Disseminated gonococcal infection (DGI) is defined as the identification of *N. gonorrhoeae* from non-mucosal surfaces like blood, CSF, skin, and synovial fluid resulting from hematogenous dissemination. The incidence of DGI, estimated to be 0.5-3% of all gonorrhea infections in the past has decreased because of the declining prevalence of gonococcal strains prone to dissemination [2]. We present a case of DGI involving the mediport catheter in a patient with active breast cancer without a prior history of gonorrhea or pelvic symptoms.

## Case presentation

A 55-year-old female presented to the emergency department with pain and a purulent discharge at her mediport catheter site for two days. She also had fever, chills, and diaphoresis for the same duration. She denied sore throat, joint pain or swelling, rash, and respiratory, abdominal, or pelvic complaints. Her past medical history included grade three triple negative invasive ductal carcinoma of the left breast diagnosed six months ago. She completed 12 cycles of chemotherapy with paclitaxel and carboplatin and was currently on immunotherapy with pembrolizumab. She achieved a complete clinical response in the breast mass. She was in a monogamous relationship and was practicing oral sex as well. She denied direct exposure of genital or oral secretion to the mediport catheter site as well as any history of gonorrhea or other sexually transmitted infections.

Upon presentation, she had tachycardia, a temperature of 103^o^F. She was diaphoretic and the mediport catheter site was tender, erythematous, and had frank purulent drainage. There was no pharyngeal erythema, joint swelling, tenderness, or rash. On cardiac auscultation, no murmur was heard. Her abdominal and pelvic examination was unremarkable. Labs showed a leukocyte count of 4.5 K/mcL with a normal differential count and 8% bands. Serum creatinine, electrolytes, and liver enzymes were unremarkable. Lactic acid was 1.3 mmol/L and C-reactive protein was 228 mg/L. Two sets of blood cultures were obtained. The mediport catheter was removed and operative cultures were sent. Vancomycin and 1 gram of ceftriaxone every 24 hours were started. Both sets of blood cultures and operative cultures from the mediport catheter site were positive for gram-negative diplococcus. The organism in the blood and operative cultures was identified by matrix-assisted laser desorption/ionization time-of-flight mass spectrometry (MALDI-TOF MS) as *N. gonorrhoeae*. The MALDI-TOF MS score for the blood isolates was reported as 2.39 and 2.20, while the wound isolate received a score of 2.01. The isolates were sent to an outside laboratory for susceptibilities. A transthoracic echocardiogram was negative for valvular vegetations. Urine polymerase chain reaction (PCR) test for *N. gonorrhoeae* and *Chlamydia trachomatis* was negative. Vancomycin was stopped and ceftriaxone was continued. The patient improved clinically, and she was discharged on ceftriaxone for a total of 10 days through a midline catheter. Susceptibility results later revealed that the blood isolate was susceptible to azithromycin, cefixime, and ceftriaxone. The susceptibility testing was performed using E-strips (ETEST®, bioMérieux SA, Marcy-l'Étoile, France), and the minimum inhibitory concentrations (MIC) to ceftriaxone was 0.008. The susceptibilities were not done for penicillin and ciprofloxacin due to high rates of resistance. The patient completed the therapy and denied relapse of symptoms on a follow-up telephone call.

## Discussion

Up to 50-80% of women infected with *N. gonorrhoeae* are asymptomatic leading to a delay in treatment as compared to men [3]. The host risk factors for DGI include female gender, pregnancy, immediate postpartum state, menses, asymptomatic mucosal infection, multiple sexual partners, low socio-economic status, and intravenous drug use. Complement deficiency, liver cirrhosis, HIV infection, systemic lupus erythematosus (SLE), splenectomy and sickle cell disease are well-known risk factors for DGI.

Both paclitaxel and carboplatin are known to cause myelosuppression, but our patient had normal cell counts with differential within the normal range. Pembrolizumab is a monoclonal antibody and acts as an immune checkpoint inhibitor by blocking the binding of the human programmed death-1 receptor to its ligand, allowing activated tumor-specific T cells to kill tumor cells. None of these medications cause defects in the complement pathway. Eculizumab, a recombinant monoclonal antibody used in the treatment of paroxysmal nocturnal hemoglobinuria and atypical hemolytic uremic syndrome is a terminal complement inhibitor and has been associated with disseminated *Neisseria* infection [4]. Our patient did not receive eculizumab for any medical condition. 

DGI is often preceded by asymptomatic mucosal infection and has a broad clinical spectrum. It can range from typical arthritis-tenosynovitis-rash to infections at unusual sites. About 70% of females with DGI lack signs of genital tract involvement. Gonococcal bacteremia is often intermittent and only half of the patients with DGI have positive blood cultures [5]. Certain strains like protein 1A serotype and those lacking protein II and arginine-uracil are more prone to cause disseminated infection in the absence of urethral symptoms which is explained by their less potent inflammatory response [6]. In the literature, there are reports of gonococcal bacteremia with unusual sites of infections including liver abscess, meningitis, and endocarditis [7-11]. This includes a case of a young female with sickle cell disease and without prior genitourinary complaints presenting as gonococcal liver abscess as well as another case of gonococcal bacteremia associated with a liver abscess in a patient without any known risk for dissemination. A case of gonococcal meningitis was reported in a young female who had SLE and was taking eculizumab for atypical hemolytic uremic syndrome. An estimated 1-2% of patients with DGI can develop endocarditis and, in rare cases, may be the sole manifestation of DGI. In addition, there has been a case of a cardiovascular implantable electronic device infection with associated bacteremia due to *N. gonorrhoeae* [12]. From our review of PubMed and Google Scholar, there are no other reported cases of mediport or other central venous catheter infections due to *N. gonorrhoeae*. As in our case, the endovascular device is presumed to have been secondarily seeded from a bacteremia originating from a mucosal surface. As our patient's urine PCR was negative for *N. gonorrhoeae*, her most likely source of infection was from the oral mucous membranes.

Antibiotics that are effective against *N. gonorrhoeae* include third-generation cephalosporins, fluoroquinolones, and penicillin. Because of increasing resistance to penicillin and ciprofloxacin, susceptibilities should be used to guide the therapy if the patient does not show a clinical response to empiric treatment. The recommended duration of treatment for uncomplicated bacteremia is 7-10 days; however, gonococcal meningitis and endocarditis require longer treatment duration. Treatment of sexual partners is an integral part of management to decrease the risk of reinfection to the patient and transmission to other people.

## Conclusions

Patients may present with DGI with an inapparent source as they may have dissemination from mucosal surfaces without localized symptoms. Our case demonstrates that not only can DGI occur from an inapparent source, but this can also result in secondary infection of endovascular devices. Prompt recognition and removal of infected endovascular devices by *N. gonorrhoeae* is required for optimal treatment.
